# Associations between stuttering, comorbid conditions and executive function in children: a population-based study

**DOI:** 10.1186/s40359-020-00481-7

**Published:** 2020-10-31

**Authors:** Ai Leen Choo, Sara Ashley Smith, Hongli Li

**Affiliations:** 1 Department of Communication Sciences and Disorders, Georgia State University, 30 Pryor St SW, Atlanta, GA 30303 USA; 2 Department of Teaching and Learning, University of South Florida, Tampa, FL 33620 USA; 3 Department of Educational Policy Studies, Georgia State University, 30 Pryor St SW, Atlanta, GA 30303 USA

**Keywords:** Stuttering, Comorbidity, Executive function, Socioemotional competence, Children

## Abstract

**Background:**

The aim of this study was to investigate the relationship between executive function (EF), stuttering, and comorbidity by examining children who stutter (CWS) and children who do not stutter (CWNS) with and without comorbid conditions. Data from the National Health Interview Survey were used to examine behavioral manifestations of EF, such as inattention and self-regulation, in CWS and CWNS.

**Methods:**

The sample included 2258 CWS (girls = 638, boys = 1620), and 117,725 CWNS (girls = 57,512; boys = 60,213). EF, and the presence of stuttering and comorbid conditions were based on parent report. Descriptive statistics were used to describe the distribution of stuttering and comorbidity across group and sex. Regression analyses were to determine the effects of stuttering and comorbidity on EF, and the relationship between EF and socioemotional competence.

**Results:**

Results point to weaker EF in CWS compared to CWNS. Also, having comorbid conditions was also associated with weaker EF. CWS with comorbidity showed the weakest EF compared to CWNS with and without comorbidity, and CWS without comorbidity. Children with stronger EF showed higher socioemotional competence. A majority (60.32%) of CWS had at least one other comorbid condition in addition to stuttering. Boys who stutter were more likely to have comorbid conditions compared to girls who stutter.

**Conclusion:**

Present findings suggest that comorbidity is a common feature in CWS. Stuttering and comorbid conditions negatively impact EF.

## Background

Disruptions in the fluent flow of speech are a hallmark of stuttering [1]. However, consequences of the disorder extend beyond speech. There is a growing body of evidence pointing to deficits in cognitive and metalinguistic skills in children who stutter [2–5]. CWS have been reported to show weaker executive function (EF; namely, phonological working memory [WM], attentional skills and inhibitory control) relative to children who do not stutter [CWNS; for a review see 6–11], with implications for fluency [12, 13]. EF is the umbrella term used to describe the abilities needed to manage and allocate cognitive resources during cognitively challenging activities, such as switching between rules or tasks, controlling and focusing attention, ignoring distractions, and inhibiting impulses [11, 14]. EF is fundamental for language, self-control, emotional regulation, and goal-oriented behaviors [15–17].

### EF in typical development

EF follows a predictable developmental timeline [18], emerging in infancy as the ability to direct attention and progressing into the complex abilities required for goal-oriented behaviors in adulthood [11, 19–21]. EF supports language development (e.g., attention facilitates language learning), and (phonological) WM supports novel vocabulary acquisition by allowing children to attend to, analyze and hold linguistic representations and rules over time [for a review see 22–27]. In preschool- and school-age children, stronger WM, attention and inhibitory control are correlated with better expressive and receptive language skills [28–31]. This association may extend beyond childhood, as both children and adults with stronger EF are more successful in learning a new language [32]. The relationship between EF and language are likely bidirectional. Language may facilitate EF performance by helping children to construct representations by labeling conditions, allowing them to reflect on and use rule structures that underlie EF tasks [33, 34]. In typically developing children, steeper vocabulary growth at age 3 years predicts EF abilities at age 5 years [25]. Higher inhibitory control is associated with greater task perseverance among children, and higher EF is positively associated with vocabulary [35]. Further evidence for the relationship between language and EF comes from children’s self-talk during EF tasks. Four- and 10-year olds who use self-talk during the Tower of London task (a commonly used measure of EF) showed faster performance and required a smaller number of moves to completion [36–38].

EF skills are also predict socioemotional competence in typically developing children [18, 39]. EF in preschool predicts social competence in kindergarten [40]. Children must use EF skills including WM (to remember social norms), inhibition (to suppress socially inappropriate responses), and attention (to direct and sustain focus) to regulate behaviors and emotions [14, 41, 42]. Deficits in WM is linked to inattentive behavior, high impulsivity, anxiety and depression in children [43–48]. Lower inhibitory control is associated with aggressive behavior, and lower social skills [18, 45–47, 49]. Notably, stronger inhibitory control, i.e., better self-regulation, is correlated with higher social status (more popular) in children [50, 51]. Attentional problems in early childhood are also correlated with delinquency, and problem conduct such as aggression and antisocial behaviors in adolescence [52–54].

EF is thought to be foundational to academic performance and success [for a review see 55–57]. Children must sustain attention, attend to important features of lessons, avoid distractions and hold information in memory in the classroom [58]. Perhaps not surprisingly, weaker EF is associated with lower academic progress, and lower teacher scores for working hard at school and learning skills [18, 35, 45–47, 49, 59]. Reading and writing skills are also subserved by EF; requiring phonological awareness, and the ability to hold, manipulate, and integrate visual, auditory and linguistic information in WM [11, 16]. Children with lower self-regulation and attentional problems show poorer reading and writing abilities [52, 53, 60, 61].

EF components while core to the development of self-regulation, socioemotional competence, and academic achievement are also crucial for fluency [62, 63]. Typically developing children and adults with higher WM capacity produce more utterances and lower rates of disfluencies (e.g., part-word repetitions, revisions) during spontaneous speech and reading compared to their peers with lower WM capacity [63–65]. Conditions of divided attention where participants perform concurrent tasks result in higher frequency of repetitions and interjections compared to non-divided attention (e.g., speech only) tasks [66]. Similarly, adults and children with lower inhibitory control show higher rates of disfluencies (e.g., revisions) during production of sentences [67].

In general, measuring EF in young children has proved difficult [68, 69]. The majority of assessments are adaptations of tests for adults, as such, children particularly those in preschool or younger, may lack the linguistic and motoric proficiency required for these tasks, resulting in floor effects [for a review see 70, 71]. Further, the issue of ecological validity of these assessments, whether they are able to capture executive functioning in real-word situations, have been challenged [72–74]. The use of validated and normed parent surveys and self-reports, such as the Behavior Rating Inventory of Executive Function (BRIEF), which measures the behavioral expression of EF provide a solution to some of these challenges [75]. Children’s behavior at home or school provide settings for observing EF capacity, and there is accumulating evidence that parent and teacher ratings of everyday, real-world behaviors in these environments provide ecologically valid assessments in children [70, 71]. EF manifests in everyday behaviors such as getting along with others (e.g., inhibitory control/socioemotional regulation), completing tasks (attention/self-regulation), and academic achievement (WM/attention) in both typically developing and clinical pediatric populations [71, 76, 77]. Deficits in EF are correlated with behaviors such as learning difficulty, inattentive behavior, poor task completion, and slower academic progress [43–47, 54]. Accordingly, questions on the BRIEF such as: “Has trouble finishing tasks (chores, homework, etc.)”, “Has trouble concentrating on tasks, schoolwork, etc.”, “Gets out of control more than friends”, and “Has trouble getting used to new situations (classes, groups, friends, etc.)” rated on a Likert scale (“N” if *the behavior is never a problem*, “S” *if the behavior is sometimes a problem*, and “O” *if the behavior is often a problem*) offer multiple perspectives on a child’s EF. Other parent surveys and self-reports such as the Child Behavior Checklist [CBCL; 78] and Strength and Difficulties Questionnaire [SDQ; 79] also offer insights into behaviors regulated by EF including socioemotional competence. The CBCL includes parent and teacher ratings *(0* = *Not true, 1* = *Somewhat or sometimes true,* and *3* = *Very true or Often True)* on questions for assessing challenges in socioemotional development such as “Worrying, Unhappy sad, or depressed”, “Doesn’t get along with other children”, and “Doesn’t know how to have fun, acts like a little adult”.

### EF and stuttering

Both parent reports and cognitive assessments have been used to evaluate EF in CWS and they suggest EF components are depressed in this population [6 for a review see 80]. WM underpins the ability to store and manipulate relevant information during complex tasks, and is proposed to be critical for fluency [64, 81–83]. Children and adults who stutter show lower performance (more errors, slower reaction time) in WM tasks (e.g., non-word repetition [NWR] and digit span tasks) compared to CWNS [e.g., 84–92]. However, WM deficits may be less evident in CWS during less complex tasks (e.g., 2- vs. 5-syllable NWR tasks), pointing to a compromised system unable to accommodate increased demands [7, 89, 93–96]. Research suggests a correlation between WM capacity, stuttering severity, and recovery [8, 97]. Close to stuttering onset, CWS who eventually recover show stronger WM compared to CWS who do not recover [8]. Additionally, CWS with lower WM capacity (indexed by higher error rates on NWR) show more severe stuttering compared to CWS with higher WM [97].

Executive attention oversees available resources for cognitive processes including speech production [98, 99]. Both direct and indirect measurements suggest greater difficulty in managing attention for CWS compared to CWNS [for a review see 80]. Parent- and teacher-reports point to lower attentional flexibility and sustained attention in CWS [100–102]. These reports are consistent with findings of slower response times compared to CWNS, and a negative correlation between accuracy and speed in CWS using direct measures of attention (e.g., Dimensional Card Change Sort, Posner Test of Covert Attention Shift) which require target selection and shifting attention toward different cues [9, 103, 104]. Weaker attention control is also correlated with higher frequency of stuttering in CWS [105, 106]. Similarly, in adults who stutter divided attention, i.e., managing concurrent tasks (e.g., speech and finger tapping), results in higher rates of stuttering [107 however, see 108]. Attentional training (using flanker tasks) have been reported to reduce stuttering severity in CWS [109]. Notably, the link between attention regulation and fluency may not be specific to stuttering. In the Felsenfeld, van Beijsterveldt, and Boomsma [102] study, both CWS and CWNS with higher rates of typical disfluencies were more likely to have attentional issues (based on parent report) compared to CWNS with lower rates of typical disfluencies. Attentional control may also have implications for recovery. Parents report shorter attention span in both CWS who recovered and CWNS compared to CWS with chronic stuttering [110], which could signal faster processing speeds or lower levels of perseveration in those who recover.

Inhibitory control underpins self-regulation and the ability to suppress interfering stimuli [62, 111, 112]. There has been growing interest in the development of inhibitory control in CWS but findings have been contradictory [for a review see 6]. Some studies using direct measures of inhibition (e.g., Go/NoGo tasks) report lower accuracy and slower reaction time in preschool- and school-age CWS compared to CWNS [9, 10, 113, 114]. However, others have failed to find differences (e.g., in the number of correct inhibitions) between CWS and CWNS using similar tasks [115]. Findings based on parent reports have been similarly varied. While some report lower inhibitory control and self-regulation in CWS relative to CWNS [116, 117], others have found similar [85, 118–121] or stronger inhibitory control [122, 123] in CWS relative to CWNS. Markedly, weaker inhibitory control in CWS is associated with more severe stuttering and chronicity [105, 124–126]. It is plausible that CWS with stronger inhibitory control may have greater ability to suppress overt expressions of incorrect speech programs resulting in lower rates of stuttering or higher probability of recovery [127].

### EF in other developmental disorders and children with comorbid conditions

Deficits in EF are frequently reported in speech-language, and neurodevelopmental disorders such as attention deficit hyperactivity disorder (ADHD) and autistic spectrum disorder [ASD; for a review see 24, 128–131]. In preschool- and school-age children, specific language impairment (SLI) is associated with weaker EF [WM, attention and inhibitory control; 130, 132]. Children with ADHD show lower performance (reflected by lower accuracy and slower response time) on tasks requiring WM, attention and inhibitory control compared to typically developing children [128, 133]. The degree of EF deficits may vary across disorders. For example, parent-ratings of children with reading disability suggests higher EF than for children with ADHD or ASD [77].

Comorbidity is commonly reported in neurodevelopmental disorders, with potential implications for EF development. Children with comorbid conditions show more profound EF deficits compared to children without comorbidity [129, 134, 135]. For example, children with multiple diagnoses of ADHD and anxiety or conduct disorders show slower completion time, higher error rates and more perseveration on EF tasks (e.g., Wisconsin Card Sorting, Finger Windows) which necessitate WM, attention and inhibitory control, relative to children with ADHD without comorbidity [136, 137]. These findings are consistent with parent reports [e.g., Behavior Rating Inventory of Executive Function (BRIEF); 75] of lower EF in children with comorbidity compared to children without comorbidity [134]. It is noteworthy that chronic health conditions are also associated with impaired EF. For example, children with medical conditions such as diabetes and sickle cell anemia show significant impairments in attention and EF tasks compared to children without those conditions [138].

The prevalence of comorbid conditions, such as learning disabilities and developmental delay, is higher for CWS relative to CWNS [139, 140]. In clinical cohorts, concomitant language, speech, and behavioral disorders (e.g., expressive language, receptive language, articulation, phonology, and ADHD) are commonly reported with stuttering [141, 142]. Prior studies also suggest higher rates of socioemotional, psychological distress and anxiety in CWS compared to CWNS [126, 143–148]. In a study of 2,628 CWS, a majority (62.8%) had comorbid disorders [149]. The most commonly reported comorbidity in CWS were learning (15.2%), reading (8.2%), attention deficit disorder (ADD, 5.9%) and behavioral disorders [2.4%; 149]. Medical diseases, such as diabetes, asthma, and sickle cell anemia have also been found at higher rates in CWS compared to CWNS [149–151]. Although CWS commonly show symptoms of other disorders, the intervening role of comorbidity on EF has not received as much attention. It is plausible that similar to children with other developmental disorders, CWS with comorbidity would show weaker EF compared to those without comorbidity.

### Present study

Findings related to EF in CWS have been ambivalent [see 6, 80]. Variability across studies may be a function of the tasks employed. CWS may perform within norm or equivalently to CWNS in less complex tasks (e.g., 2-string forward digit span) but show lower performance in more complex EF tasks (e.g. Dimensional Change Card Sort, backward digit span). In other words, deficits in EF (as a function of impairment or developmental timeline) may not be evident unless the system is sufficiently taxed; for example, involve EF domains which have not fully developed (attentional control in 3-year olds), or necessitate manipulation or transformation of information (e.g., Backward Digit Span). Findings from a study examining performance accuracy across multiple EF tasks in 602 typically developing preschool children between 3 and 6 years may shed some insight on ambivalent reports in CWS [152]. Carlson [152] found that performance was dependent on task complexity, whereby, outcomes (i.e., behavioral accuracy) were similar for tasks with equivalent levels of difficulty regardless of the task design (e.g., requiring a motor or verbal response). For example, 4-year olds show comparable accuracy on two tasks with equivalent complexity levels which tap into different EF domains: Whisper (inhibition: children must inhibit from shouting out names of cartoon characters but instead whisper them), and Motor Sequencing [WM: imitate sequence of pressing keyboard from left to right with index finger as fast as possible before the experimenter says “Stop”; 152]. However, these same 4-year olds showed poorer performance on the more complex Day/Night task where children must suppress the prepotent response, recall the correct answer, and generate a new response which conflicts with the dominant (say “night” for the sun picture, and “day” for moon picture). Tasks which tap into multiple EF domains (e.g., Dimensional Change Card Sort and Backward Digit Span which require both WM and inhibitory control) were found to be more difficult [152]. Collectively, these findings suggest comparing across studies utilizing disparate tasks will likely result in ambivalent findings. Studies which employ less demanding tasks may lack the sensitivity to detect EF differences between CWS and CWNS.

Notably, a study by Ntourou, Anderson and Wagovich [153] reported better sensitivity for detecting differences in EF between CWS and CWNS using an indirect measure, i.e., the BRIEF parent report [75]. CWS received lower parent ratings for WM, inhibitory control, and attentional control compared to CWNS [153]. Further, the likelihood of CWS meeting the clinically significant criteria for EF difficulties were 2.5 to 7 times higher than for CWNS. CWS also received particularly low ratings on questions related to behaviors involving a combination of WM, inhibitory control/self-regulation and attention: “Has trouble finishing tasks such as games, puzzles, pretend play activities”, “Reacts more strongly to situations than other children”, and “Resists change of routine, food, places, etc*.*”. In contrast, a direct behavioral measure, Head–Toes–Knees–Shoulders (HKTS—which also involves WM, inhibitory control and attention) failed to detect differences between CWS and CWNS. Findings from this study point to the validity and sensitivity of behavioral manifestations to detect EF deficits in CWS.

The aim of this study was to investigate the relationship between EF, stuttering, and comorbidity by examining CWS and CWNS with and without comorbid conditions. To do this, we examined behaviors (such as inattention, self-regulation including emotional and social regulation, and task completion) underpinned by or closely associated with EF using a population-based data. Based on previous findings in CWS and CWNS, we hypothesize that: (1) CWS will show weaker EF compared to CWNS, (2) children with comorbid conditions will show weaker EF compared to children without comorbid conditions, and (3) children with stronger EF will also show higher socioemotional competence compared to children with weaker EF.

## Methods

### Sample

Data was accessed from the National Health Interview Survey (NHIS) from years 2006–2018. The NHIS is a nationally administered cross-sectional survey, conducted by the Centers for Disease Control and Prevention (CDC) to monitor the health of the U.S., including trends in illness and disabilities [154]. The survey has been administered annually since 1957, providing a nationally representative sample of households in all 50 states and the District of Columbia. For each household, data was collected from a randomly selected sample adult and child. Information about the child was collected from an adult, typically the parent or guardian. Data was collected face-to-face by trained interviewers who read questions on the survey to interviewees. Some segments of the population were excluded including U.S. citizens not residing in the country, active duty military personnel, incarcerated inmates, and long-term care facility patients. A total of 119,983 children (girls = 58,150; boys = 61,833) were sampled between 2006 and 2018.

### Identification of CWS and CWNS

CWS were identified with a positive parent response, “Yes”, to the question “During the past 12 months, has [SC^1^] had any of the following conditions: Stuttering or stammering”. Other possible responses were: “No”, “Refused”, “Not ascertained” or “Don’t know”. CWNS were identified by a “No” response to “Stuttering or stammering”.

*Comorbidity.* CWS and CWNS were further distinguished into groups with and without comorbidity based on parent report. Children with one or more comorbid conditions were identified as CWS-WC and CWNS-WC. Whereas CWS and CWNS without any comorbid condition were grouped as CWS-NC and CWNS-NC respectively. Comorbidity was determined by a “Yes” response by parents to one or more of the following questions: “Has a doctor or health professional ever told you that [SC^1^] had _____” related to (1) “Attention Deficit Hyperactivity Disorder (ADHD) or Attention Deficit Disorder (ADD)”, (2) “Down syndrome”, (3) “Cerebral palsy”, (4) “muscular dystrophy”, (5) “Cystic fibrosis”, (6) “Sickle cell anemia”, (7) “Autism”^2^, (8) “Diabetes”, (9) “Ever told SC had arthritis”, (10) “Congenital heart disease”, (11) “Other heart condition”, and (12) “Asthma”. Other possible responses to these questions were: “Refused”, “Not ascertained” or “Don't know”.

### EF

EF was identified on the basis of parent responses to the following questions: (1) “Well behaved/does what requested, past 6 m”^3^, (2) “Good attention/completes chores, homework, past 6 m”^4^, and (3) “Difficulties w/emotions/concentration/behavior/getting along” (see Table 1). For questions 1 and 2, possible responses were “Not true”, “Somewhat true”, “Certainly true”, “Refused”, “Not ascertained”, or “Don’t know”. For question 3, possible responses were “No”, “Yes, minor difficulties”, “Yes, definite difficulties”, “Yes, severe difficulties”, “Refused”, “Not ascertained” or “Don't know”. Responses that did not provide estimates of EF, i.e., “Refused”, “Not ascertained” or “Don't know” were excluded from the analysis. Other responses were assigned scores to reflect the level of executive functioning. For questions (1) and (2), responses were assigned the following scores: 1 = “Not true”, 2 = “Somewhat true”, 3 = “Certainly true”. For question (3), responses were assigned the following scores: 1 = “Yes, severe difficulties”, 2 = “Yes, definite difficulties”, 3 = “Yes, minor difficulties”, and 4 = “No”. An aggregate score with a maximum value of 10 (high EF) and minimum value of 3 (low EF) based on these questions were used in the analyses.

**Table 1:** Questions related to executive function (EF) on the NHIS, and equivalent items on other surveys

Executive function		
NHIS	BRIEF-P [75]	BASC—EF [155]
Well behaved/does what requested, past 6 m	Gets out of control more than friends; Has outburst for little reason; Acts too wild or “out of control” (on the Teacher form)	Acts out of control; Listens to directions
Good attention/completes chores, homework, past 6 m	Has short attention span; Has trouble finishing tasks (chores, homework, etc.)	Pays attention; Has short attention span; Is easily distracted
Difficulties w/emotions /concentration/ behavior/getting along	Has explosive angry outburst; Has trouble concentrating on tasks, schoolwork, etc.; Reacts more strongly to situations than other children, Becomes upset too easily	Has trouble concentrating

*BASC* Behavior Assessment System for Children, *BRIEF* Behavior Rating Inventory of Executive Function—Parent Rating Scales, *NHIS* National Health Interview Survey

### Socioemotional competence

To determine whether socioemotional competence was also correlated with EF as previously reported in typically developing children, responses to the following questions were included in the analysis: (1) “Many worries/often seems worried, past 6 m”, (2) “Unhappy/depressed/tearful, past 6 m”, and (3) “Gets along better w/adults than children/youth, past 6 m”^5^ (see Table 2). Responses that did not provide estimates of socioemotional competence, i.e., “Refused”, “Not ascertained” or “Don't know” were excluded from the analysis. Other responses were scored: 3 = “Not true”, 2 = “Somewhat true”, 1 = “Certainly true”. A composite score with a maximum of 9 (high socioemotional competence) and minimum of 3 (low) were possible.

**Table 2:** Questions related to socioemotional competence on the NHIS, and equivalent items on other surveys

Socioemotional competence		
NHIS	CBCL [78]	SDQ [79]
Many worries/often seems worried, past 6 m	Worries	Many worries, often seems worried
Unhappy/depressed/tearful, past 6 m	Cries a lot; Unhappy, sad, depressed	Often unhappy, down-hearted or tearful
Gets along better w/adults than children/youth, past 6 m	Doesn’t get along with other kids; Compared to others of his/her age, how well does your child: Get along with his/her brothers & sisters? Get along with other kids? Behave with his/her parents?	Gets on better with adults than with other children

*CBCL* Child Behavior Checklist, *NHIS* National Health Interview Survey, *SDQ* Strength and Difficulties Questionnaire

### Data analyses

To combine the NHIS data from 2006 to 2018, we adjusted the weights and stratum according to the NHIS guidelines. Descriptive statistics based on the sample population, using SPSS version 25 [156], were used to describe the distribution of stuttering and comorbidity across group and sex. Subsequent regression analyses to test the three hypotheses were conducted with Mplus 8.0 [157], accounting for complex sampling design of the NHIS so that results are representative of the US population. For hypothesis 1 and 2, the dependent variable was EF, and for hypothesis 3 the dependent variable was socioemotional competence. The predictors included comorbid status (0 without comorbidity, 1 with comorbidity), and sex (0 if male, 1 if female).

## Results

### Prevalence of stuttering and comorbid conditions

A total of 2258 CWS (girls = 638, boys = 1620), and 117,725 CWNS (girls = 57,512; boys = 60,213) aged between 3 and 17 years were identified in the sample. The overall prevalence of stuttering was 1.88% with a male-to-female ratio of 2.54:1 (Table 3). There was a higher prevalence of stuttering (4.19%) and male-to-female ratio (2.86:1) for children with comorbid conditions relative to children without comorbid conditions (1.02%; 2.14:1; Table 3). A majority (60.32%) of CWS had at least one other comorbid condition in addition to stuttering compared to CNWS where less than a third (26.44%) had one or more conditions.

**Table 3:** Prevalence and male-to-female ratio of stuttering for children with and without comorbid conditions

Comorbidity status	Sex	CWS		CWNS		Prevalence of stuttering (%)
		*N*	M:F ratio	*N*	M:F ratio	
Without comorbid conditions	Male	611	2.14:1	41,308	0.91:1	1.02
	Female	285		45,287		
With comorbid conditions	Male	1009	2.86:1	18,905	1.55:1	4.19
	Female	353		12,225		
Total	Male	1620	2.54:1	60,213	1.05:1	1.88
	Female	638		57,512		

*CWS* children who stutter, *CWNS* children who do not stutter, *M:F* male:female, *N* sample size

For both CWS and CWNS, ADHD, asthma and autism were the most prevalent comorbid conditions (Table 4). Across both groups, rates of comorbidity were higher for males compared to females.

**Table 4:** The prevalence and number of conditions in the sample population

Condition	CWS			CWNS		
	Male (%)	Female (%)	Total (%)	Male (%)	Female (%)	Total (%)
ADHD/ADD	520 (29.80)	135 (18.72)	655 (26.56)	7420 (11.52)	3203 (5.21)	10,623 (8.43)
Asthma	221 (46.72)	82 (45.30)	303 (46.33)	3869 (34.05)	2964 (36.59)	19,462 (35.11)
Autism	195 (11.17)	61 (8.45)	256 (10.38)	1475 (2.29)	409 (0.66)	1884 (1.49)
Other heart conditions	50 (2.86)	23 (3.18)	73 (2.95)	579 (0.90)	537 (0.87)	1116 (0.89)
Cerebral palsy	39 (2.23)	23 (3.18)	62 (2.51)	367 (0.57)	310 (0.50)	677 (0.54)
Downs Syndrome	23 (1.31)	8 (1.11)	31 (1.25)	86 (0.13)	75 (0.12)	161 (0.13)
Congenital heart disease	9 (0.51)	6 (0.83)	15 (0.61)	127 (0.20)	134 (0.22)	261 (0.21)
Diabetes	8 (0.46)	4 (0.55)	12 (0.49)	151 (0.23)	158 (0.26)	309 (0.25)
Sickle cell anemia	8 (0.46)	2 (0.28)	10 (0.40)	92 (0.14)	91 (0.15)	183 (0.15)
Arthritis	6 (0.34)	5 (0.69)	11 (0.45)	48 (0.074)	91 (0.148)	139 (0.110)
Muscular dystrophy	4 (0.23)	4 (0.55)	8 (0.32)	21 (0.033)	19 (0.031)	40 (0.032)
Cystic fibrosis	0	0	0	24 (0.037)	21 (0.034)	45 (0.036)

*ADD* attention deficit disorder, *ADHD* attention deficit/hyperactivity disorder, *CWS* children who stutter, *CWNS* children who do not stutter

### Prediction of EF by stuttering and comorbidity (Hypotheses 1 and 2)

Table 5 shows the mean EF across stuttering status (CWS vs CWNS), comorbidity status (with or without) and sex. EF was lower in CWS compared to CWNS, children with comorbid conditions relative to children without comorbidity, and boys compared to girls.

**Table 5:** Weighted means and standard deviations for executive function (EF) composite scores across stuttering status (with and without stuttering), comorbidity status (with and without comorbidity) and sex

Comorbidity	Sex	CWS			CWNS		
		*M*	*SD*	*N*	*M*	*SD*	*N*
Without comorbid conditions	Male	7.683	2.358	611	8.331	2.285	41,308
	Female	8.018	2.115	285	8.457	2.296	45,287
With comorbid conditions	Male	6.254	2.444	1009	7.324	2.472	18,905
	Female	6.539	2.361	353	7.747	2.435	12,225

*CWS* children who stutter, *CWNS* children who do not stutter, *M* mean, *N* sample size, *SD* standard deviation

Table 6 summarizes the results of the regression analysis with EF as the dependent variable. As shown in Model 1, stuttering was a significant predictor of EF (*B* = − 1.195, *p* < .001). EF was significantly lower for CWS compared to CWNS, supporting Hypothesis 1—CWS will show weaker EF compared to CWNS.

**Table 6:** Results of the regression analyses showing the contributions of stuttering, comorbidity and sex to executive function (EF)

Models	Predictor	Unstandardized		Standardized		*p* value	*R*^2^
		*B*	*SE*	*β*	*SE*		
Model 1	Intercept	8.161	0.012	3.426	0.016	< .001	.006
	Stuttering	− 1.195	0.076	− 0.077	0.005	< .001	
Model 2	Intercept	8.390	0.013	3.522	0.017	< .001	.031
	Comorbidity	− 0.950	0.023	− 0.176	0.004	< .001	
Model 3	Intercept	8.401	0.013	3.527	0.017	< .001	.035
	Stuttering	− 0.947	0.073	− 0.061	0.005	< .001	
	Comorbidity	− 0.920	0.023	− 0.171	0.004	< .001	
Model 4	Intercept	8.094	0.030	3.398	0.020	< .001	.037
	Stuttering	− 0.920	0.073	− 0.060	0.005	< .001	
	Comorbidity	−0.893	0.023	− 0.166	0.004	< .001	
	Sex	0.201	0.018	0.042	0.004	< .001	

Models 1 and 2: Effect of each predictor on EF score, Model 3: Stuttering and comorbidity entered in a single model, and Model 4: Model 3 additionally adjusting for sex

*B* unstandardized regression coefficient, *β* standardized regression coefficient, *SE* standard error

As shown in Model 2, comorbid conditions was also a significant predictor of EF (*B* = − 0.950, *p* < .001). The EF for children with comorbidity was significantly lower than for children without comorbidity, supporting Hypothesis 2—Children with comorbidity will show lower EF compared to children without comorbidity.

In Model 3, stuttering and comorbidity remained significant when entered concurrently. Given the potential differences in EF between boys and girls, sex was included in Model 4. Females had slightly higher EF than males (*B* = 0.201, *p* < .001). In summary, Hypotheses 1 (CWS < CWNS) and 2 (with comorbidity < without comorbidity) were confirmed (see Table 5).

### Prediction of socioemotional competence by EF (Hypotheses 3)

Table 7 shows the mean socioemotional competence scores across stuttering, comorbidity status, and sex. Socioemotional competence was lower for CWS compared to CWNS, children with comorbidity compared to children without comorbidity, and girls compared to boys.

**Table 7:** Weighted means and standard deviations for socioemotional competence composite score across stuttering status (CWS and CWNS), comorbidity status (with and without comorbidity) and sex

Comorbidity	Sex	CWS			CWNS		
		*M*	*SD*	*N*	*M*	*SD*	*N*
Without comorbid conditions	Male	7.795	1.429	519	8.226	1.357	36,038
	Female	7.940	1.316	250	8.114	1.430	39,354
With comorbid conditions	Male	7.096	1.833	883	7.740	1.597	16,591
	Female	7.143	1.693	311	7.673	1.575	10,672

*CWS* children who stutter, *CWNS* children who do not stutter, *M* mean, *N* sample size, *SD* standard deviation

Table 8 summarizes the results of the regression analysis with socioemotional competence as the dependent variable. As shown in Model 1, children with higher EF had significantly higher socioemotional competence, supporting Hypothesis 3—Children with stronger EF will also show higher socioemotional competence compared children with weaker EF. When EF was increased by 1, socioemotional competence increased by 0.453 (*β* = 0.512, *p* < .001).

**Table 8:** Results of regression analyses showing the contributions of executive function (EF), stuttering, comorbidity and sex to socioemotional competence

Models	Predictor	Unstandardized		Standardized		*p* value	*R*^2^
		*B*	*SE*	*β*	*SE*		
Model 1	Intercept	4.017	0.060	2.722	0.055	< .001	0.262
	EF	0.453	0.007	0.512	0.006	< .001	
Model 2	Intercept	8.097	0.019	5.488	0.047	< .001	.000
	Sex	− 0.041	0.012	− 0.014	0.004	< .010	
Model 3	Intercept	8.050	0.007	5.456	0.045	< .001	.005
	Stuttering	− 0.694	0.070	− 0.073	0.008	< .001	
Model 4	Intercept	8.162	0.008	5.532	0.045	< .001	.020
	Comorbidity	− 0.476	0.015	− 0.143	0.005	< .001	
Model 5	Intercept	4.294	0.065	2.910	0.059	< .001	0.266
	EF	0.457	0.007	0.516	0.006	< .001	
	Stuttering	− 0.112	0.055	− 0.012	0.006	.042	
	Sex	− 0.201	0.011	− 0.068	0.004	< .001	
	Comorbidity	− .019	0.014	− 0.006	0.004	.180	

Models 1–4: Effect of each predictor on socioemotional score, and Model 5: All predictors entered in a single model

*B* unstandardized regression coefficient, *β* standardized regression coefficient, *SE* standard error

To control for other relevant factors, sex, stuttering status, and comorbidity status were included individually (Models 2–4) and concurrently (Model 5) in the analyses. As shown in Model 5, sex had a significant (*B* = − 0.201, *p* < .010) but small effect on socioemotional competence; scores were slightly higher for males than females. Stuttering (*B* = − 0.694, *p* < .001) and comorbidity (*B* = − 0.476, *p* < .001) status had significant negative effects on socioemotional competence. Socioemotional competence score was lower for CWS compared to CWNS (Model 3) and lower for children with comorbidity compared to children without comorbidity (Model 4).

In Model 5, when all predictors were entered in a single step, EF remained statistically significant. This further confirmed hypothesis 3, i.e., children with stronger EF will show higher socioemotional competence. Stuttering status remained a significant predictor (*B* = − 0.11, *p* = .042), although the effect was smaller than in Model 3. Sex was still significant (*B* = .041, *p* < .001), but with a larger effect than in Model 2. However, comorbidity status was no longer significant (*B* = − 0.019, *p* = 0.18) after inclusion of the other variables in the model.

## Discussion

The aim of this study was to investigate the relationship between EF, stuttering, and comorbidity. To the best of our knowledge, this is the first study to examine EF in CWS with and without comorbidity on a large scale. Our findings point to a critical association between stuttering, comorbidity, and EF in both CWS and CWNS. First, weaker EF was correlated with having stuttering. Second, having comorbid conditions was also associated with weaker EF. Notably, CWS-WC showed the weakest EF among all groups of children. Third, higher socioemotional competence was associated with stronger EF and absence of stuttering. Our study also confirmed expected epidemiological trends on a large scale. We present evidence for a higher prevalence of stuttering and higher male-to-female ratio in children with comorbidity.

### Prevalence of stuttering

The overall prevalence of stuttering was consistent with past reports [for a review see 1, 158–160]. Closer inspection of the data indicates higher rates of stuttering in children with comorbidity, particularly boys, compared to children without comorbid conditions.

#### Comorbidity

A majority of CWS had comorbid conditions in the present study, consistent with previous studies [e.g., 161–163]. Similar to past studies, ADHD and asthma were two of the most frequently reported comorbid condition in CWS [149, 164, 165]. Interestingly, ADHD has been identified as a risk factor for stuttering [166]. Several explanations have been offered to explain the high rates of comorbidity with other neurodevelopmental disorders in stuttering. First, stuttering and other neurodevelopmental disorders are thought to share a core deficit or similar risk factors [e.g., ADHD; 167], and as such, CWS could be at higher risk for developing other disorders and vice versa [168]. Second, stuttering may represent one outcome along a continuum of (common or overlapping) etiologies and disorders, with variability across severity, timing, and symptoms [169]; and children with comorbidity may represent a more severe end of the continuum. Alternatively, stuttering may be a distinct disorder that negatively impacts development, amplifying susceptibility to other disorders [170].

The high rates of asthma in CWS in the present study is in agreement with past reports [143, 149, 151, 171]. In fact, another atopic disease, hay fever, was reported to correlate with an earlier onset of stuttering and chronicity [172]. The inflammatory response associated with atopic diseases is thought to affect the neurocircuitry including those involved in speech [172, 173]. Markedly, adults with asthma show atypical gray [e.g., increased gray matter volume in the right superior temporal gyrus; 174], and white [e.g., lower white matter coherence in the inferior frontal gyrus; 175] matter in regions involved in speech production and reported to be affected in stuttering [176]. Although the mechanism of causality is unclear, the relationship between atopic diseases and stuttering, suggests that research on the impact of childhood health outcomes and stuttering is warranted. Overall, the higher rates of comorbid conditions may be a corollary of symptoms that manifest more severely in CWS, reaching observable or clinical levels. In general, screenings and treatment across multiple conditions may be necessary in a majority of CWS.

#### Sex differences

Present findings also point to sex as a significant variable in susceptibility to stuttering and comorbid conditions. Overall, there was a higher male-to-female ratio of stuttering in this sample, a finding in line with the sexually dimorphic nature of this disorder. This sex bias has been attributed to increased vulnerability among males, i.e., a lower “stuttering threshold” and/or fewer required contributing factors to developing stuttering, compared to females where greater loading is required [177, p. 21]. Another proposed explanation is that differences in cognitive maturation and development between sexes might result in more severe manifestation of symptoms in males. According to this theory, females are equally at risk for stuttering, however, symptoms manifest less severely or below clinical levels [178]. In the current study, the male-to-female ratio was higher for CWS-WC relative to CWS-NC. This greater sex bias for CWS-WC compared to CWS-NC suggests increased vulnerability for males who stutter. It is worth mentioning that the preponderance of affected males is not limited to stuttering. Other disorders, such as autism and ADHD, show similar trends of greater male susceptibility [179, 180]. It has also been suggested, however, that sex differences are due to discrepancies in diagnosis. For example, ADHD is more likely to be diagnosed in boys [181] and it is unclear if this is rooted in differences in ADHD presentation [i.e., boys may present in a manner such that diagnosis is more likely; 182] or actual differences in prevalence [for evidence of similar presentation between sexes, for example 183]. It is beyond the scope of the present paper to determine the mechanisms underlying this bias, specifically, if they are rooted in differences in prevalence or differences in diagnosis. We suggest this as a direction for future research; understanding the combination of these factors would not only inform how stuttering and comorbid disorders manifest, but also translate into optimal treatment for each sex.

### Predictors of EF

Stuttering, comorbidity status, and sex were found to predict EF scores. Consistent with our hypothesis, CWS showed weaker EF compared to CWNS although the magnitude of the difference was relatively small. Specifically, CWS received lower parent ratings for statements addressing behaviors (see “EF” section in Methods) that necessitate holistic EF, WM, attention, and inhibitory control. Taken together, findings from the current and past studies suggest that weaker EF may be a feature of stuttering [e.g., 10, 85, 102].

There is accumulating evidence that EF is mediated by a wide network of circuity, with the (pre)frontal cortices and basal ganglia playing key roles [for overview see 184–186]. The (pre)frontal cortex is involved in manipulating and transforming information held in WM [187 involving Brodmann area [BA] 44–47, 188, 189]; inhibiting prepotent behavioral and neural responses, and activating representations in subcortical regions [190, 191]; and top-down control of attention, i.e., bias attention to relevant information, and sustaining attention [192–195]. EF behaviors localized in the (pre)frontal regions are modulated by activity in the basal ganglia, which select and enable executive programs [184, 196–199]. These same regions, (pre)frontal cortex and basal ganglia, have been found to be aberrant in stuttering [200, 201 for overview see 202]. It is highly plausible that EF deficits in CWS are related to these structural and functional abnormalities.

In typically developing children, EF components experience protracted development from infancy through late childhood and into early adulthood [for a review see 69, 203]. Although many EF components are present in infancy, they grow exponentially in early childhood [16, 26, 55, 97, 204–207]. Children show limited ability to manipulate or transform representations in WM until around age 2 years [208]. Before age 4 years, children perform below chance on inhibitory control tasks [e.g., Grass/Snow or Less is More; 152, 209]. The ability to sustain and direct attention to relevant stimuli are limited until about age 5 years [210, 211]. Presentation of neurodevelopmental disorders and stressors in early life have particularly profound impact on EF development [212, 213]. The developmental timing of stuttering, with onset typically around 3 years of age [214], may have devastating effects on EF during this critical period of rapid growth. The presence of stuttering may delay, reduce or plateau EF development. A longitudinal study which maps EF growth would be needed to determine specific trajectories in CWS.

Previous studies have primarily focused on EF differences between CWS without comorbidity and typically developing children [85–87, 89]. The present study extended this focus to CWS and CWNS with comorbidity. Although the magnitude of difference between groups were small, our findings of weaker EF in CWS and CWNS with comorbidity compared to their peers without comorbid conditions is consistent with prior reports of weaker EF in children with multiple conditions in other neurodevelopmental disorders (see “EF in other developmental disorders and children with comorbid conditions” section).

Nonetheless, our finding of stronger EF in CWS-NC compared to CWNS-WC was unexpected. Additionally, CWS-WC showed the weakest EF amongst all groups. These findings suggest that multiple conditions have a more robust negative effect on EF than stuttering alone, and further widen disparities between CWS and CWNS. A potential confound to understanding the effects of comorbidity is the severity of conditions, duration and sequence of appearance. In the present study, it is unclear whether stuttering is the core impairment in CWS-WC, and whether conditions occurred sequentially (and if so, in which order). Moreover, the duration of overlap between conditions was not reported. It is plausible that CWS (and for that matter, CWNS) with early onset or longer duration of multiple diagnoses would show weaker EF as a consequence of prolonged, increased burden. To gain a better understanding of the possible causal influences and directionality of stuttering, comorbidity and EF, a longitudinal study mapping the sequence, timing, and duration of conditions in conjunction with EF development along varying pathways to recovery or chronicity would be necessary. It should be noted that the standardized regression coefficient for comorbidity was larger compared to that for stuttering. This suggests that the presence of comorbid conditions may have a larger impact on EF development than stuttering alone.

The present study also found a significant association between sex and EF. When stuttering and comorbidity were controlled for, the EF for females was larger than the EF for males. Nonetheless, the magnitude of difference between sexes was small. In fact, the standardized regression coefficient for stuttering was larger compared to that for sex. This suggests that stuttering may have a greater practical importance than sex in determining EF. Nonetheless, prior research has demonstrated differences between the sexes for specific EF components during childhood [215], although differences lessen with age [216] and there is no evidence of systematic advantage across the lifespan [for a review see 217]. In general, typically developing girls outperform boys on inhibitory control and attention [217]. Girls are also less impulsive during childhood and show better WM [217], although differences are not observed on tasks of spatial WM [218, 219]. Additionally, within-sex variability is likely greater than between-sex variability [217]. The current study addressed holistic EF measured through parent report of behavior; prior research indicates that sex differences are sometimes linked to EF task type, such that changing task features changes results in turn [217]. As such, findings of sex differences may be related to EF measurement in the current study (i.e., parent report of behaviors) and should be interpreted with caution.

### Predictors of socioemotional competence

EF was a significant predictor of socioemotional competence, confirming our hypothesis. The standardized regression coefficient for EF was larger than that for sex or comorbidity status pointing to the crucial contribution of EF to socioemotional development. Stronger EF was correlated with better socioemotional competence, a finding in line with general consensus in the field. Social interactions involve EF skills, including the ability to remember social norms (WM), suppress socially inappropriate behaviors (inhibitory control), and direct and sustain attention on interactions [14, 18, 220]. Accordingly, children with stronger EF would be expected to have better socioemotional functioning and more prosocial behaviors. Socioemotional competence is a key predictor of social and academic success, and challenges with socioemotional functioning in early childhood have consequences for long-term social, academic success and mental health [221–223]. Early socioemotional competence in kindergarten is correlated with lower probability of mental health issues in adolescence; and higher probability of graduating from high school, attending college, being employed in adulthood [222, 224]. Conversely, lower socioemotional competence in preschool is linked to higher internalizing (e.g., depressed mood, anxiety, social withdrawal) and externalizing (e.g., aggression, hyperactivity) symptoms in adolescence [225]. It is worth noting that these same challenges in social, academic and mental health are reported in those who stutter [226–228], and multifactorial models of stuttering cite emotion as a factor in the emergence and chronicity of stuttering [229, 230]. However, the cross-sectional design of the current study does not allow for the examination of EF changes and related socioemotional competence over time in CWS. Moreover, other factors found to impact socioemotional status in children, such as socioeconomic status/household income, language minority status and parents’ mental health [224], were not examined in this study. A longitudinal study which encompasses the aforementioned factors would be needed to provide a complete picture of socioemotional functioning in CWS.

Sex was also found to be a significant predictor of socioemotional competence. When EF, stuttering, and comorbidity were controlled for, girls were found to have lower socioemotional competence than boys, although the magnitude of difference was small. This suggests that the effects of sex may be less clinically significant than EF or comorbidity in the development of socioemotional competence. Disparities in sex-related findings between EF and socioemotional competence may be related to differences in social-evaluative concerns, perceptions of socioemotional competence between sexes, and the measures of socioemotional competence used in the present study. Girls have been found to show heightened socio-evaluative concerns compared to boys, as well as higher levels of depression related to these concerns [231]. Further, girls who show lower socioemotional competence exhibit higher externalizing symptoms [232]. Traditionally, perceptions of socioemotional competence may be impacted by the expected norms for sexes, where externalizing behaviors such as aggression are judged favorably in boys (i.e., aggressive boys are seen as more socially competent than less aggressive boys) but not girls [for overview see 233]. These differences may intersect with the specific items used in the current study, that is, higher levels of depression, worrying, and externalizing behaviors among girls may have disproportionate impact on parent ratings on the related survey items.

Stuttering was also a significant predictor of socioemotional competence, although the magnitude of effect was smaller than for EF or sex. This finding was not surprising in light of reports of social and emotional difficulties in those who stutter [147, 234]. School-aged CWS between 7 and 12 years old, particularly girls, are six times more likely to have social anxiety disorder, and seven times more likely to have generalized anxiety disorder compared to typically developing children [235]. An overwhelming majority of CWS experience peer victimization, difficult in establishing friendships, negative self-perceptions, shame, and lower self-confidence with consequences for their socioemotional functioning [236–240]. Collectively, findings point to the burden of stuttering on socioemotional functioning, particularly for girls who stutter.

### Theoretical implications for EF in stuttering

EF components feature prominently in some causal theories of stuttering, such as the EXPLAN model, Covert Repair Hypothesis and Vicious Circle Hypothesis [13, 108, 241–243]. In the EXPLAN model, the phonological loop and WM are involved in accessing phonological information in memory, and lags between linguistic planning and motor execution are thought to produce disfluencies [12, 13, 244]. It is conceivable that deficits in phonological WM (as reflected by lower accuracy and slower response in WM tasks in CWS) could result in errors in activation or ordering of linguistic material, and result in linguistic planning delays [12, 245]. The Covert Repair Hypothesis proposes that disfluencies are the product of covert detection and corrections of prearticulatory errors which interfere with ongoing articulation, and higher rates of disfluencies are due to multiple or excessive attempts at repairs [13]. First, weaker attention control as reported in CWS [e.g., 9, 10, 113, 114] may result in excessive attention on prearticulatory errors or an inability to shift attention away from repaired segments, whereby, numerous repair attempts are made, contributing to high rates of disfluencies. Second, weaker inhibitory control would also prevent suppression of excessive corrections of speech plans, yielding high rates of disfluencies [127]. Similarly, the Vicious Circle Hypothesis posits that heightened monitoring and focus on speech errors, along with lower threshold for repairs underpin stuttering [108]. Reports of weaker attention control and lower flexibility in CWS [e.g., 100–102] could result in abnormal allocation of resources or an inability to redirect attention away from error monitoring. These theories posit a link between stuttering frequency and EF development. CWS with weaker EF would be predicted to show higher frequency of stuttering. These frameworks could also be extended to stuttering prognosis. For children who are experiencing development delays including in EF domains, stuttering may resolve as the cognitive system matures and catches up. Delays in phonological access may decrease as WM capacity increases [EXPLAN; 12]. As inhibitory control strengthens, attempts at repairs may decline [Covert Repair Hypothesis; 13], and stronger attention control could reduce excessive monitoring of speech errors [Vicious Circle Hypothesis; 243].

WM models offer a unified framework for integrating EF components affected in stuttering. The *unity*/*diversity* theoretical model of EF proposes that underlying components (WM, attention, inhibition) are correlated but dissociable [112]. EF holistically results from the interaction of these distinct domains with each responsible for complimentary control [112, 246]. Baddeley and Hitch’s [247] *three-factor* model and more recently, Baddeley’s [98] *four-factor* model conceptualize WM as a storage system for verbal/auditory information (i.e., phonological loop), and visuo-spatial information (i.e., visuospatial sketchpad). The phonological loop and visuospatial sketchpad are overseen by an attention controller (i.e., central executive), and episodic buffer for integration of material in the phonological and visuospatial subsystems [98, 248, 249]. There is robust evidence which suggest that the WM system (including attention and inhibitory control) is crucial for EF behavior, and deficits within this system in whole or within each domain, are correlated with behavioral issues [43, 250 for a review see 251]. Thus, the development of EF may related to specific behaviors (e.g., self-regulation) in CWS. In other words, CWS with behavioral challenges, including those related to self- and socioemotional regulation, and attention may have weaker EF. Nonetheless, how EF maps onto chronicity is unclear. It is plausible that CWS with EF deficits, reflected by behavioral challenges, that do not resolve with age may have a higher risk for chronicity.

## Limitations

The strengths of the present study are the large sample sizes and avoidance of potential selection bias of subjects (e.g., recruitment via clinical referrals). Nonetheless, some limitations including the reliance on parent report need to be addressed. It is extremely probable that stuttering was not formally diagnosed in some children and others were misidentified. Further, parents’ memory of their child’s development including stuttering may be inaccurate. The heterogeneity of stuttering and variability of symptoms over time may also inflate the risk of misidentification, particularly for children with mild stuttering or those who may be experiencing periods of increased fluency at the time of the survey interview. EF was also measured using parent reports. Although previous studies have utilized behavior as a proxy for EF in CWS [e.g., 100, 102, 116] and CWNS [e.g., 70, 71, 75]; and found higher sensitivity for detecting differences between CWS and CWNS [153], more research is need to determine how parent reports map onto outcomes in standardized EF tests for CWS. Stuttering was not operationally defined in the survey. Thus, CWNS with higher rates of typical disfluencies may be misidentified with stuttering. The presence of comorbidity were also based on parent reports, and as such may be disproportionately (over- or under-) identified. Although parents were asked whether “a doctor or health professional ever told” them that their child had specific conditions, it is unclear if these were based on a formal diagnosis. Additionally, the direction of effects or causality between factors cannot be determined in the current study. The current study included children with a broad range of comorbid conditions. Future studies may benefit from examining the impact of specific conditions on EF development.

## Conclusion

Findings from the present study points to the validity and sensitivity of parent reports on real-world behaviors as a means to measure EF in CWS. Nonetheless, it is still unknown if EF is malleable in CWS, and if so, what are the opportunities for remediation, such as targeted training, and/or authentic activities that support EF development [cf. musical training, mindfulness; 252]. Managing two languages concurrently is thought to enhance EF components in typically developing bilinguals [for a review see 253]. Bilinguals show higher performance for tasks requiring WM, attention and inhibitory control compared to their monolingual peers [253–255 however, see 256]. For example, 4-year old bilinguals outperform their age-matched monolingual peers in their capacity to focus and switch attention, demonstrating accuracy equivalent to 5-year old monolinguals during the Dimensional Change Card Sort [257]. It is unclear if this bilingual enhancement is affected in stuttering. Understanding how stuttering interacts with bilingualism could offer insight into the development of EF in CWS.

Current findings support the presence of subtypes in CWS based on EF and comorbidity, i.e., CWS with stronger EF without comorbid conditions, and CWS with weaker EF with comorbidity. These subtypes may have relevance for chronicity. Some children who are experiencing developmental delays, including in cognitive development, may experience periods of stuttering, that is, until EF deficits resolve with maturation of the cognitive system. It is possible that stronger EF, albeit weaker than in CWNS, and the absence comorbidity in CWS reflect more subtle deficits which could eventually resolve or attenuate. If so, the probability of recovery would be higher for this group. Conversely, CWS with weaker EF and comorbid conditions may be a subgroup with greater developmental vulnerability and increased risk for chronicity. Weaker EF and comorbidity may simply signal a higher degree of impairment, surpassing the ability of the cognitive system to compensate. More fine-grained research is needed to disentangle the relationship between EF, comorbidity, and stuttering prognosis. Present findings also have implications for clinical practice. Deficits in EF and high rates of comorbidity in CWS underscore the need for multi-dimensional, multi-domain approaches to the diagnoses and treatment of stuttering. Such an approach would better address the complexity of stuttering, and variability across individuals and sex across a wide spectrum of symptoms leading to improved outcomes.

## Data Availability

The dataset used for this study was obtained from the National Health Interview Survey, and available from the CDC https://www.cdc.gov/nchs/nhis/index.htm.

## Abbreviations

ADD: Attention deficit disorder
ADHD: Attention deficit hyperactivity disorder
ASD: Autistic spectrum disorder
BA: Brodmann area
BASC: Behavior Assessment System for Children
BRIEF: Behavior Rating Inventory of Executive Function
CBCL: Child Behavior Checklist
CDC: Centers for Disease Control and Prevention
CWNS: Children who do not stutter
CWNS-NC: Children who do not stutter without comorbidity
CWNS-WC: Children who do not stutter with comorbidity
CWS: Children who stutter
CWS-NC: Children who stutter without comorbidity
CWS-WC: Children who stutter with comorbidity
EF: Executive function
HKTS: Head–Toes–Knees–Shoulders
NHIS: National Health Interview Survey
NWR: Non-word repetition
SC: Selected child
SDQ: Strength and Difficulties Questionnaire
SLI: Specific language impairment
WM: Working memory

## Footnotes

1 SC refers to selected child.

2 After 2010, this question was reworded to “Ever told SC had autism/autism spectrum disorder”, and from 2014 onwards this question was reworded to “Ever told SC had autism, Asperger's, pervasive developmental disorder or autism spectrum disorder”.

3 Question 1 was not available for the 2008 and 2009 surveys.

4 Question 2 was not available for the 2008 and 2009 surveys.

5 Questions 1, 2 and 3 were not available for 2008 and 2009 surveys.
